# A Case Study into Microbial Genome Assembly Gap Sequences and Finishing Strategies

**DOI:** 10.3389/fmicb.2017.01272

**Published:** 2017-07-18

**Authors:** Sagar M. Utturkar, Dawn M. Klingeman, Richard A. Hurt, Steven D. Brown

**Affiliations:** ^1^Graduate School of Genome Science and Technology, University of Tennessee Knoxville, TN, United States; ^2^Biosciences Division, Oak Ridge National Laboratory Oak Ridge, TN, United States; ^3^BioEnergy Science Center Oak Ridge, TN, United States

**Keywords:** PacBio, Illumina, genome assembly, next-generation sequencing (NGS), repetitive DNA, Pilon, circlator

## Abstract

This study characterized regions of DNA which remained unassembled by either PacBio and Illumina sequencing technologies for seven bacterial genomes. Two genomes were manually finished using bioinformatics and PCR/Sanger sequencing approaches and regions not assembled by automated software were analyzed. Gaps present within Illumina assemblies mostly correspond to repetitive DNA regions such as multiple rRNA operon sequences. PacBio gap sequences were evaluated for several properties such as GC content, read coverage, gap length, ability to form strong secondary structures, and corresponding annotations. Our hypothesis that strong secondary DNA structures blocked DNA polymerases and contributed to gap sequences was not accepted. PacBio assemblies had few limitations overall and gaps were explained as cumulative effect of lower than average sequence coverage and repetitive sequences at contig termini. An important aspect of the present study is the compilation of biological features that interfered with assembly and included active transposons, multiple plasmid sequences, phage DNA integration, and large sequence duplication. Our targeted genome finishing approach and systematic evaluation of the unassembled DNA will be useful for others looking to close, finish, and polish microbial genome sequences.

## Introduction

Since the first Next-Generation Sequencing (NGS) platform was released by 454 Life science (Margulies et al., 2005), there has been a remarkable increase in sequencing efficiency, throughput, and read lengths (Koren and Phillippy, 2014). Sequencing costs continue to drop dramatically and whole genome sequencing is within reach for small-scale laboratories on relatively modest budgets. During the past decade, the sequencing industry has been largely dominated by the second generation, sequencing by synthesis platforms such as Illumina which are characterized by the low-cost, high-throughput, and short reads with high accuracy (van Dijk et al., 2014). Short sequencing reads have limited power to resolve large repetitive regions even within small microbial genomes (Chain et al., 2009; Nagarajan and Pop, 2013). Short read technologies are generally able to resolve microbial genomes up to the high-quality draft standard (Treangen and Salzberg, 2012), which is sufficient for many applications such as understanding gene-coding potential, strain typing, or pan-genome analysis (Roberts et al., 2013). However, draft genomes are fragmented assemblies that can contain misassembled regions, incorrect gene calls, and other artifacts. Fragmented assemblies are often attributed to repetitive DNA regions (such as rRNA operons) which are abundant in microbial genomes and present the greatest technical challenge to the assembly process especially when the repetitive region is longer than the read lengths (Treangen and Salzberg, 2012; Brown S. et al., 2014). Finished genome sequences are high quality by definition, represent more accurate genomic information and are often desirable for model organisms and industrially important microbes (Fraser et al., 2002; Thomma et al., 2015).

The application of new protocols (e.g., use of complementary paired and mate-pair libraries) and algorithm developments have facilitated improved genome assemblies. Progress in next-generation sequencing platforms, metrics, and performances has been reviewed (Liu et al., 2012; Quail et al., 2012; van Dijk et al., 2014), assessments for various assembly methods conducted (Salzberg et al., 2012; Magoc et al., 2013; Koren and Phillippy, 2014; Utturkar et al., 2014), and various applications (Buermans and den Dunnen, 2014; Rhoads and Au, 2015) have been discussed elsewhere. Development of so-called third-generation sequencing platforms for single-molecule sequencing is a more recent development for producing long sequence reads which facilitate assembly. Pacific Biosciences (PacBio) RS-II instrument outputs are characterized by long reads and average read lengths are reported in the range of 10–11 kb (Hua and Hua, 2016). Relatively high rate of random errors within individual reads can be overcome by error-correction algorithms given sufficient sequencing depth (Chin et al., 2013). The longest reported PacBio reads from the RS-II instrument extend well beyond 20 kb. A key aspect of longer reads is their ability to span large repetitive regions, which greatly aids the assembly process (Brown S. et al., 2014; Koren and Phillippy, 2014; Utturkar et al., 2015) when sufficient coverage (>100x) is available (Chin et al., 2013; Koren et al., 2013). In 2014, Oxford Nanopore Technologies released a nanopore-based sequencer for long single molecule DNA reads (Feng et al., 2015). In the time since its release, hybrid and *de novo* assembly strategies have also been developed and tested using Oxford Nanopore datasets (Risse et al., 2015; Deschamps et al., 2016).

The application of longer sequencing reads facilitated finished genome assemblies for many bacterial genomes (Koren et al., 2013). The utility of long reads is demonstrated by the increasing number of finished genomes obtained using PacBio technology (Koren et al., 2013; Brown S. D. et al., 2014; Eckweiler et al., 2014; Harhay et al., 2014; Mehnaz et al., 2014; Satou et al., 2014; Kanda et al., 2015; Nakano et al., 2015). However, examples exist where genomes are only resolved into 10 or fewer contigs despite high (>100x) PacBio sequence coverage (Hoefler et al., 2013; Dunitz et al., 2014; Bishnoi et al., 2015; Okutani et al., 2015; Shapiro et al., 2015; The NCTC 3000 Project, 2016), and manual finishing is necessary to obtain complete genome sequences. Substantial developments for long read assembly methods and analysis are reported, but information is lacking on the nature of unassembled DNA regions or gaps within unfinished PacBio assemblies. Therefore, a systematic evaluation of draft, near-finished (containing up to 10 contigs) and finished genome assemblies would be useful to reveal the features and properties of the unassembled DNA regions from Illumina and/or PacBio platforms.

In the present study, seven bacterial genomes were sequenced using Illumina Paired-End (PE) and PacBio RS-II platforms. *De novo* and hybrid genome assemblies were created using platform specific or hybrid datasets from Illumina and PacBio platforms with various assembly programs and parameter optimizations. In this focused study, manual genome finishing was performed for two genomes, generating up to finished grade assemblies and permitted further analysis of prior gap sequences for which there is a dearth of data. Additional genome polishing was performed on PacBio assemblies with the recently described Pilon software (Walker et al., 2014). The impact of improving genome assemblies and polishing was assessed by several metrics that included gene models. This study offers insights into the nature of gaps associated with Illumina and PacBio assemblies of microbial genomes, describes bioinformatics and manual steps for assembly improvement and underlines the importance of post-assembly polishing steps for genome refinement.

## Materials and methods

### Whole genome sequencing

Whole genome sequencing data for seven microorganisms (*Clostridium pasteurianum* ATCC 6013 (Pyne et al., 2014), *Clostridium paradoxum* JW-YL-7 (Lancaster et al., 2016), *Clostridium thermocellum* AD2 (Utturkar et al., 2016), *Pelosinus fermentans* UFO1 (Brown S. D. et al., 2014), *P. fermentans* JBW45 (De Leon et al., 2015), *Halomonas* sp. KO116 (O'Dell et al., 2015) and *Bacteroides cellulosolvens* DSM 2933) (Dassa et al., 2015) using Illumina MiSeq (Illumina, San Diego, CA, USA) (Quail et al., 2012) and PacBio RS-II (Pacific Biosciences, Menlo Park, CA, USA) (Korlach et al., 2010) platforms have been reported. The bacteria were chosen for the availability of Illumina and PacBio sequence data, with most having relevance to bioenergy applications, and in the case of *P. fermentans* species they are fermentative metal-reducing bacteria. For all genomes in current study, Illumina paired-end library preparation, PacBio SMRTbell library preparation, and sequencing protocols are performed as described previously (Utturkar et al., 2015). GenBank and SRA sequence accession numbers for each genome are provided in Table S1.

### Data quality control, genome assembly, and annotation

Quality based trimming of raw Illumina data was performed using CLC Genomics workbench software (CLC) to remove bases having PHRED quality score <30 and any reads shorter than 20 bp. Adapter trimming and filtering of raw PacBio data was performed through SMRT analysis software to obtain “filtered subreads” with default parameters (Utturkar et al., 2015). *De novo* genome assembly of Illumina data was performed using SPAdes version 3.5.0 (Bankevich et al., 2012) and ABySS version 1.5.2 (Simpson et al., 2009) with parameter optimization (Utturkar et al., 2014). Hybrid assembly of Illumina and PacBio data was performed using SPAdes hybrid assembler version 3.5.0 with default parameters. Exact commands used for SPAdes and ABySS assemblies are provided in Section S1. Long read PacBio data were assembled using the SMRT Analysis software and the HGAP protocol (Chin et al., 2013). In the HGAP protocol, the “Target Coverage” parameter was updated to 15X as recommended for microbial genomes (Pacific-Biosciences, 2014a). The specific versions of SMRT Analysis software used for each genome are provided in the results section. Assembly summary statistics were determined using Quast software version 2.3 (Gurevich et al., 2013). Gene-calling and genome annotation were performed through the Prodigal algorithm and microbial genome annotation pipeline at Oak Ridge National Laboratory (Hyatt et al., 2010; Woo et al., 2014).

### Manual genome finishing

Manual genome finishing was performed using bioinformatics tools and PCR/Sanger sequencing. During bioinformatics steps, contigs from different draft and hybrid genome assemblies were mapped to PacBio-only assemblies using Geneious software version 8.1.6 (Biomatters, Auckland, New Zealand) (Kearse et al., 2012) with default parameters. Mapping results were manually inspected to identify a possible extension (or overhang) relative to reference contigs. Supported extensions were added to the reference contigs and assembly of contigs (super-assembly) was created through Geneious software to derive a longer consensus sequence. See Section S1 for details of the Geneious software modules used in each step. Bioinformatically derived contig extensions and super-assembly derived consensus sequences were verified by PCR and Sanger sequencing. Bioinformatics finishing steps, designing of PCR/Sanger sequencing based validations and various experimental modifications of standard PCR protocol are described in detail in Section S1 with examples of two manually finished genomes (Figures S1, S2, and S3).

### Analysis of unassembled (gap) DNA

Mapping of Illumina draft contigs to finished/near-finished assemblies was performed using the “Map to Reference” module in the Geneious software, followed by manual inspection to reveal Illumina gaps and associated annotations. PacBio gaps were revealed through manual finishing of two genomes and the resulting sequences were submitted to the mfold web server (Zuker, 2003) to determine DNA folding properties and secondary structures. Default DNA folding parameters in mfold software were modified to mimic the PCR conditions (folding temperature = 55^0^ C, [Na^+^] concentration = 50 mM, [Mg^++^] concentration = 2.5 mM). Positional preference was determined using PerPlot and PerScan tools (Mrazek et al., 2011) with default parameters, and genes with periodicity intensity cutoff higher than 2.5 were determined.

### Post-assembly polishing and validation steps

PacBio-only assemblies were polished by running one additional round of the Quiver algorithm (Chin et al., 2013), followed by basecall correction through Pilon software (Walker et al., 2014) (version 1.13) with default parameters. Quiver uses PacBio reads while Pilon uses Illumina reads to perform base corrections and derive an accurate consensus sequence. The circular nature of HGAP derived contigs was assessed via the dot-plotting tool Gepard (Krumsiek et al., 2007) and circular genome sequences were derived through an alignment approach described in PacBio training manual (Pacific-Biosciences, 2015). The presence of non-chromosomal DNA such as a plasmid or phage-DNA elements was tested by evaluation of any singleton sequences and/or “deg.fasta” files (which may contain high copy number sequences such as plasmids or phage DNA) generated during the HGAP protocol. For assemblies containing fewer than 5 contigs, each contig was individually tested for circularity. The presence of plasmid DNA was further analyzed by searching for the annotated plasmid related genes such as “RepA—plasmid replication protein.” Additionally, DNA base modification analysis was performed for complete genomes using SMRT analysis software and methylation profiles (Pacific-BioSciences, 2014b) were determined for incorporation into the REBASE database (Roberts et al., 2015). REBASE is a database for information on recognition and cleavage sites for both restriction enzymes and methyltransferases and methylation sensitivity. PacBio data generates data on modified bases, which may be useful for related studies. Pilon corrections and comparison of Illumina and PacBio assemblies were further assessed by measuring the impact of nucleotide changes on protein coding potential and positive/negative influence on gene calling accuracy (See Section S1 for details).

## Results and discussion

### Sequencing and assembly overview

Illumina sequence coverage for each genome is >200X, sufficient to derive high-quality draft genome assemblies (Haridas et al., 2011; Utturkar et al., 2014). PacBio sequence coverage for each genome is >100X except for the isolates of *Pelosinus* sp. UFO1 (97x) and *B. cellulosolvens* DSM 2933 (48X). Post-trimming and filtering statistics for Illumina and PacBio data including the number of reads, average read lengths and genome coverage and total bases are summarized in Tables S1, S2, respectively. Genome assemblies were performed using combinations of Illumina and PacBio platforms and various assembly programs. Consistent with previous results (Brown S. et al., 2014), most of the genomes in the current study have superior PacBio-only assemblies (based on assembly statistics) followed by hybrid and Illumina-only assemblies, respectively. Out of seven genomes, three were assembled as complete circular chromosomes, manual finishing was performed for two genomes and remaining two were reported as near-finished assemblies. Details of the assembly results and manual finishing approaches are described in later sections. Using these seven genomes as a case study, we describe the best practices to obtain high-quality genome assembly using long sequence reads, post-assembly polishing steps, and gap-closure strategies for automated near-finished assemblies. The finishing approach outlined in this study includes the use of super-assemblies and supporting Illumina data to determine contig order followed by PCR and Sanger sequencing to validate contig joining. Post-finishing data were used to determine the characteristics of the unassembled DNA regions within Illumina and PacBio assembly.

### Unassembled DNA regions in PacBio-only assemblies

Inspection of unassembled DNA regions within PacBio assemblies was performed using five gap sequences generated through manual finishing of *C. thermocellum* AD2 and *B. cellulosolvens* DSM 2933 genomes. The unassembled DNA from PacBio assemblies were analyzed for GC content, read coverage, gap length, ability to form strong secondary structures, and corresponding annotations (Table 1). GC content of gap sequences does not diverge markedly from the genome sequence. Four of five PacBio gaps were associated with lower than the recommended coverage for HGAP assembly (100x). Gaps AD2_overlap1, AD2_Gap1 and BC_Gap1 were the most difficult to resolve by PCR and Sanger sequencing and had low sequence coverages (36X, 82X, and 4X, respectively), while high average sequence coverage values were present across the genomes (see Section S1 for details).

**Table 1:** Properties of gap sequences present within PacBio assembly.

**Organism**	**Region name**	**Start**	**Stop**	**Length (bp)**	**PacBio read coverage**	**%GC**	**Corresponding annotation**
*Clostridium thermocellum* AD2	AD2_Overlap1	3,502	5,535	2033	36x	39.4	Membrane protein insertase
	AD2_Overlap2	180,557	182,612	2055	116x	35.1	Transposase DDE domain
	AD2_Gap1	558,824	559,892	1068	82x	39	Transposase mutator type
*Bacteroides cellulosolvens* DSM 2933	BC_Overlap1	6,343,204	6,349,991	6788	36x	32.5	Transposase Tn3 family protein
	BC_Gap1	6,389,652	6,390,057	405	4x	35.5	RNA-binding protein

*Average sequence coverage and GC contents of the final genome assembly are provided in the Table S2*.

**Figure 1:**
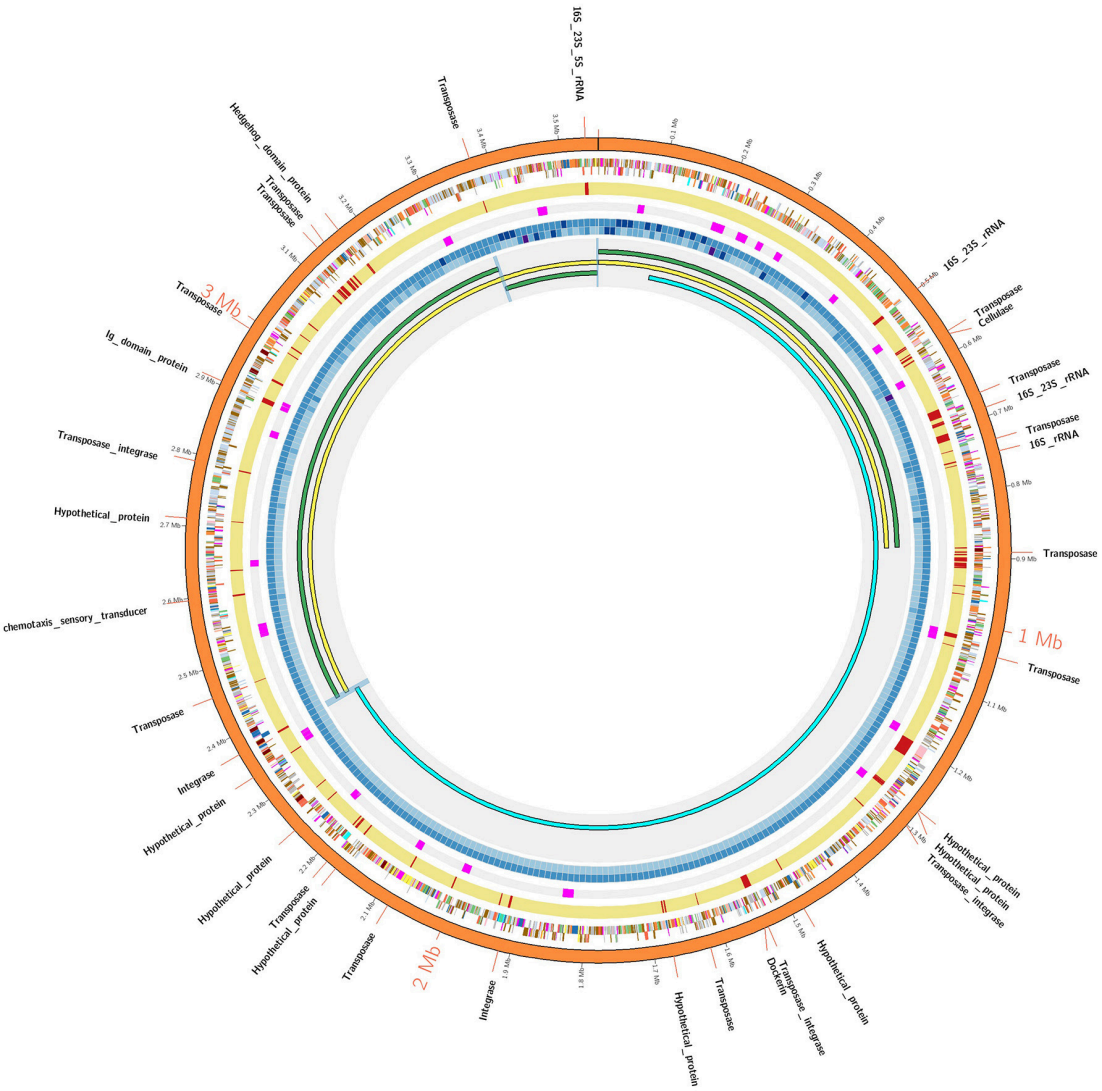
AD2 genome assembly comparisons. The outermost orange colored circle corresponds to finished genome assembly. The next two circles show genes on positive and negative strands and using color coded by standards for COG categories. The next yellow colored circle corresponds to Illumina assembly and gaps within Illumina assembly are denoted by red strokes. The next circle denotes the strong positional preference marked in pink color. The next two concentric circles denote the sequence coverage for Illumina and PacBio technologies respectively as heatmap (lowest: light blue, highest: dark blue). The innermost circle: AD2_SC1 (yellow) was generated by super assembly of draft contigs (green). AD2_HC1 (sky blue) share 780 kb overlap with AD2_SC1. Blue-highlighted region denotes sequence overlaps validated using PCR/Sanger approach. A detailed Illustration is provided in Figure S1.

Considering the low sequence coverage values and challenges associated with PCR amplification for the closed gap sequences, we hypothesized that PacBio gap sequences might form strong hairpin loop structures that would prevent DNA polymerase from being able to unwind and extend through the DNA region. To test our hypothesis, structural properties of gap sequences were analyzed using the mfold web server, which predicts the secondary structures or ability to form hairpin loops and associated minimum free energy (ΔG) values. Mfold analysis of PacBio gap sequences revealed the potential to form small stem-loop structures but large and/or strong secondary structural loops that might interfere with DNA polymerase and result in low sequence coverage were not identified. Significant differences were not observed between minimum free energies and secondary structures of PacBio gaps and 20 randomly selected regions from the AD2 and DSM 2933 genomes (Table S3). In addition, we utilized DNA periodicity criteria to determine any associations between PacBio gaps and other structural features of DNA. Regular spacing of short runs of A or T nucleotides with DNA helical period of ~10.5 bp (termed as a positional preference) has been associated with DNA curvature, supercoiling and nucleosome positioning. Relatively rigid sections of the prokaryotic DNA (characterized by short intrinsically bent DNA segments) are proposed to be associated with strong periodic patterns while structurally flexible regions are associated with weak periods (Mrazek et al., 2011; Tong and Mrazek, 2014). Positional preference was determined for all the genomes in current study and regions which correspond to Illumina gaps and also have positional preference higher than 2.50 are highlighted in orange color (Tables S4–S9). However, gaps appear to be randomly distributed as compared to strong/weak positional preference and a specific trend was not observed for this metric. An example of positional preference locations and Illumina/PacBio gaps in AD2 genome is presented (Figure 1). Post-finishing, we determined Illumina reads have uniform coverage across the gap regions. However, short read length and repetitive nature of these regions may have prevented the accurate assembly. Therefore, our initial hypothesis that resilient PacBio gaps resulted from the inability of DNA polymerase to sequence through strong hairpin loop structures was rejected.

For further characterization, we analyzed 1 kb DNA sequences flanking PacBio gaps (i.e., contig termini regions) from three near-finished genomes in this study. A self-blast was performed using 1 kb regions as a query against the entire genome using Geneious software with default parameters. The grade score from Geneious software (i.e., a cumulative score generated by combining the % pairwise identity, % query coverage, e-value) for the top blast hits for gap termini regions are described in Table S10. In each genome, except AD2, sequences flanking the gap regions showed high similarity (grade: >95%) with another region within the same genome indicating repetitive DNA sequences could have contributed to assembly challenges. Sequences flanking AD2_Gap1 have a low (grade: 72%) similarity score within the genome, consistent with the finding that the AD2 was comparatively easier to finish using standard PCR/Sanger sequencing approaches. To further validate this observation, we repeated the flanking DNA sequence analysis steps for an independent dataset (Koren et al., 2013). In three incomplete genome assemblies, most of the sequences flanking the gaps were determined to have high similarity (grade: >95%) within the same genome (Table S14) that may contribute to the fragmented PacBio assemblies.

**Figure 2:**
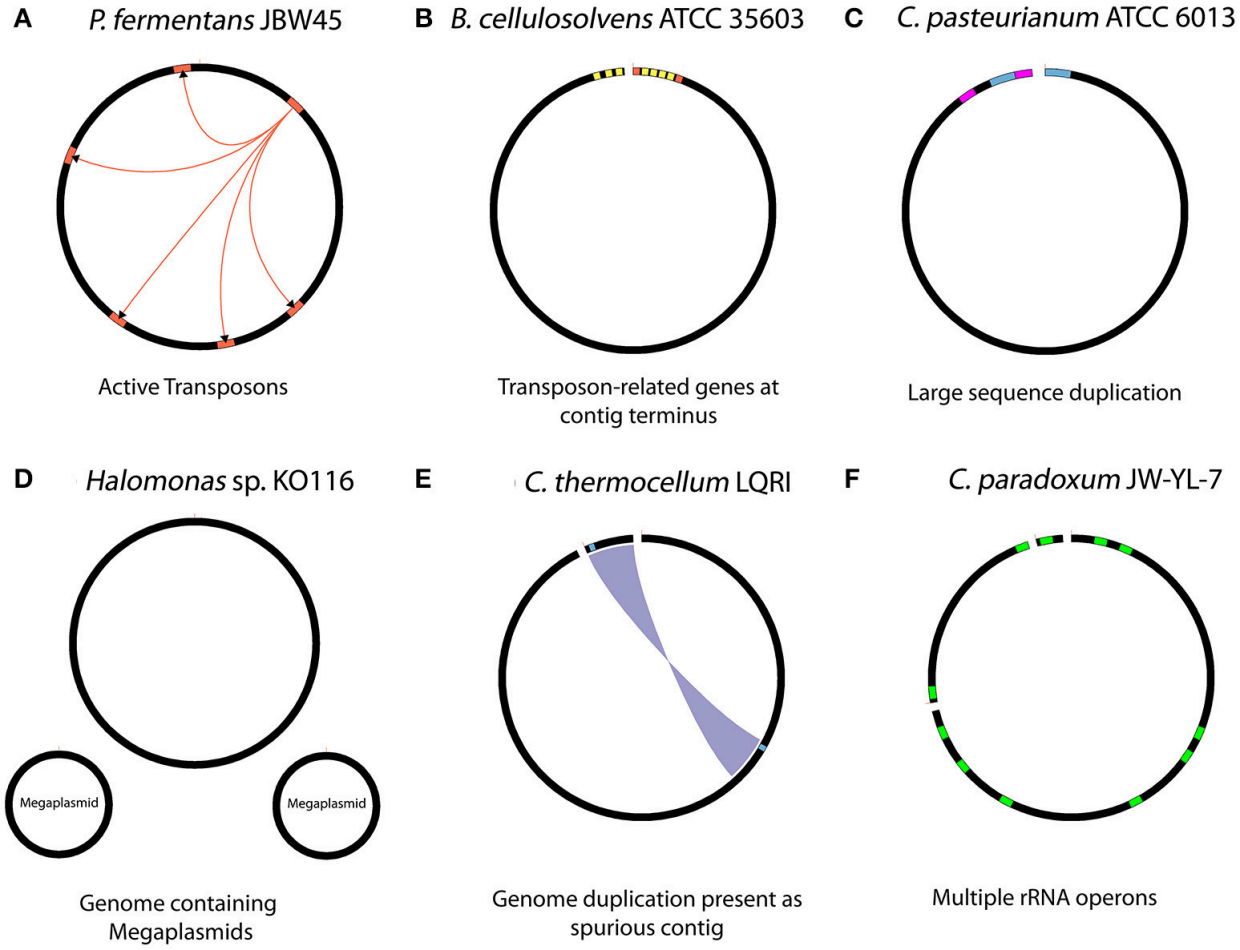
Summary of biological features with potential to interfere with the assembly process. **(A)** Presence of active transposon elements in strain JBW45 **(B)** repetitive transposon sequences at the contig terminus region of *B. cellulosolvens* **(C)** large sequence duplications in *C. pasteurianum* **(D)** presence of megaplsmids in stain KO116 **(E)** genome duplication assembled as spurious contig in *C. thermocellum* LQRI **(F)** multiple copies of rRNA operons in *C. paradoxum* JW-YL-7. The figures are illustration only and not drawn to scale.

Various biological aspects of seven genomes within this study, as well as for the *C. thermocellum* LQRI (LQRI), and *P. fermentans* DSM 17108 genomes (Utturkar et al., 2016) were further analyzed for gaps within PacBio assemblies. Specific biological features of the genomes that likely interfered with overall assembly process are summarized in Figure 2. The complete genome sequence of strain JBW45 was characterized by the presence of an active transposon element which interfered with the genome circularization process (De Leon et al., 2015). The *C. paradoxum* genome was reported to contain multiple rRNA operons with heterogeneous intervening sequences (15 different sequences in the variable region I) of 16S rRNA (Rainey et al., 1996) which could contribute to the fragmented assembly. Assembly analysis of strain ATCC 6013 and *P. fermentans* DSM 17108 revealed a possible phage integration and presence of large sequence duplication. The KO116 genome was characterized by the presence of two megaplasmids. For a previously uncharacterized bacterium, multiple contigs could lead to the impression of having a near-finished genome assembly instead of megaplasmid sequences in the absence of manual inspection. We expect new tools such as plasmidSPAdes (Antipov et al., 2016) will be useful in assembling and assessing plasmid DNA content from whole genome sequencing data. Automated HGAP assembly of LQRI obtained a near-finished assembly containing two contigs. After careful evaluation, one of the contigs was found to represent a complete circular genome while smaller 12 kb contig was determined as duplicated sequence artifact. *B. cellulosolvens* DSM 2933 contig termini were characterized by the presence of transposon-related genes (Dassa et al., 2015). In summary, our initial hypothesis that structural features of DNA (hairpin-loops, secondary structures, supercoiling, and nucleosome positioning) might affect the PacBio coverage in certain regions leading to assembly gaps was not accepted. In terms of assembly, it is likely that the HGAP software did not have sufficient read coverage to support automatic closure of these sequences and resulted in assembly gaps. Analysis of gap sequences revealed that in many cases, DNA sequences flanking the gaps have more than one copy within the genome, and some were corresponding to long repetitive elements such as “Transposon-related proteins” (De Leon et al., 2015). Further analysis revealed specific biological features such as the presence of active mobile genetic elements, plasmid sequences and phage integration which can lead to fragmented PacBio assemblies. Hence, although we could not determine one specific trend, PacBio gaps sequences were associated with a cumulative effect of number of repeats and their sizes, sequence depth, and various biological features associated with specific genomes.

### Unassembled DNA regions in illumina-only assemblies

Unassembled DNA or assembly breakpoints in Illumina assemblies were revealed by mapping the contigs from Illumina-only assembly against the final (finished or near-finished) genome assemblies. Short reads from Illumina technology have limited power to resolve longer repetitive regions (Salzberg et al., 2012; Utturkar et al., 2014) and rRNA operons are considered among the most difficult regions to assemble (Brown S. et al., 2014). Our comparisons demonstrate at least half the total rRNA operons were completely missing (unassembled) from the Illumina assembly while from remaining half, most could only be assembled partially (i.e., missing one of the 5S, 16S, or 23S elements). These findings are consistent with our previous results suggesting that rRNA operons correspond to many of breakpoints within short-read assemblies (Brown S. et al., 2014; Utturkar et al., 2014). On the other hand, longer PacBio reads resolved the majority of rRNA operons as evident through circular genome assemblies and comparison of finished vs. draft assemblies (Tables S4–S9). The total number of rRNA operons present in each genome, number of missing rRNA operons, and number of partially assembled rRNA operons in Illumina assembly are provided in Table 2. Illumina-only assemblies often also lacked tRNA due to their physical linkage to incomplete rRNA operons, as well as other genes encoding putative functions for transposase and hypothetical proteins. The average size of rRNA operons is ~5–7 kb and constituted the longest gaps within Illumina assemblies. Apart from rRNA operons, other regions that contributed to fragmented Illumina assemblies include transposon sequences, ABC-type transporters (which number in the double digits for most genomes), RNA-directed DNA polymerases (which have long sequences and share high homology), as well hypothetical proteins. A complete table describing the draft vs. finished assembly comparison details (gap coordinates, length, associated annotation, and locus tags) for each genome are provided (Tables S4–S9) and graphical representation of Illumina/PacBio gaps within AD2 genome is shown (Figure 1). The genome of *C. pasteurianum* was the only exception where two large contigs from Illumina assembly were accurate and contained all the rRNA operons and no other gaps were detected.

**Table 2:** Summary of rRNA operons present within Illumina assembly.

**Organism**	**Total rRNA operons**	**Number (percentage) of rRNA operons missing (unassembled) from Illumina assembly**	**Number of partially assembled rRNA operons in Illumina assembly**
*Clostridium thermocellum* AD2	4	2 (50)	2
*Halomonas* sp. KO116	6	4 (66)	2
*Pelosinus* sp. UFO1	14	12 (85)	2
*Pelosinus fermentans* JBW45	9	5 (55)	4
*Clostridium paradoxum* JW-YL-7	12	11 (90)	1
*Bacteroides cellulosolvens* DSM 2933	8	4 (50)	4
*Clostridium pasteurianum* ATCC 6013	10	0 (0)	0

### Insights into assembly and polishing improvement approaches

A variety of assembly algorithms are available for *de novo* and hybrid assembly (Salzberg et al., 2012; Magoc et al., 2013; Koren and Phillippy, 2014), read error correction (Lin and Liao, 2015), scaffolding (Bashir et al., 2012; English et al., 2012), and genome finishing (Swain et al., 2012) with different NGS data types. Our aim was to perform an assessment of gaps rather than an evaluation of assemblers and we chose SPAdes and ABySS to assemble Illumina data and HGAP to assemble PacBio data based on previous success (Brown S. et al., 2014; Utturkar et al., 2014). Consistent with previous findings (Brown S. D. et al., 2014), PacBio-only assemblies have the best statistics followed by hybrid and Illumina-only assemblies. Assembly summary statistics for *de novo* and hybrid assemblies are described in Table 3. It is worth mentioning that using the latest versions of assembly algorithms had significant impacts on overall assembly statistics. For example, *B. cellulosolvens* DSM 2933 (Dassa et al., 2015) and *C. pasteurianum* ATCC 6013 (Pyne et al., 2014) genomes assembled through SMRT analysis v2.2 obtained substantial improvement over v2.0 assembly (Table 3). The field of bioinformatics is rapidly evolving with the novel, efficient assembly algorithms such as Canu (Koren et al., 2017), HINGE (Kamath et al., 2017) for long reads, and integrated pipelines (Coil et al., 2015; Page et al., 2016) for short reads. For future assembly projects, it is recommended to use multiple assembly programs to obtain the optimal assembly and use our rRNA analysis approach for an additional verification of assembly accuracy (Utturkar et al., 2014). It is also important to perform a careful analysis of contigs to check for the presence of plasmid content, which could be misinterpreted as near-finished assemblies.

**Table 3:** Assembly summary statistics for *de novo* and hybrid assemblies.

**Organism**	**NGS technology**	**No. of contigs**	**Maximum contig size (kb)**	**N50 (kb)**	**Genome size (Mb)**	**Software**
*Clostridium thermocellum* AD2	Illumina	102	331	116	3.48	SPAdes^*^
		107	282	84	3.54	ABySS
	Illumina + PacBio	14	2,270	2,270	3.57	SPAdes
	PacBio-only	10	982	891	3.49	SMRTanalysis v 2.2
	PacBio-only	**1**	**3,554**	**3,554**	**3.55**	**Manual Finishing**
*Halomonas* sp. KO116	Illumina	110	373	194	5.13	SPAdes^*^
		120	315	115	5.19	ABySS
	Illumina + PacBio	30	4,654	4,654	5.19	SPAdes
	PacBio-only	**1 (+ 2)^a^**	**4,649**	**4,649**	**4.65 (+ 0.51)^a^**	**SMRTanalysis v 2.2**
*Pelosinus* sp. UFO1	Illumina	175	1,025	637	5.13	SPAdes
		131	169	78	5.03	ABySS^*^
	Illumina + PacBio	147	4,498	4,498	5.19	SPAdes
	PacBio-only	**1**	**5,115**	**5,115**	**5.12**	**SMRTanalysis v 2.1^b^**
*Pelosinus fermentans* JBW45	Illumina	70	477	244	5.3	SPAdes^*^
		114	318	110	5.4	ABySS
	Illumina + PacBio	1	5,381	5,381	5.38	SPAdes
	PacBio-only	**1**	**5,381**	**5,381**	**5.38**	**SMRTanalysis v 2.2**
*Clostridium paradoxum* JW-YL-7	Illumina	661	293	121	2.23	SPAdes
		43	235	74	1.84	ABySS^*^
	Illumina + PacBio	612	1,061	323	2.26	SPAdes
	PacBio-only	**3**	**1,855**	**1,855**	**1.93**	**SMRTanalysis v 2.2**
*Bacteroides cellulosolvens* DSM 2933	Illumina	194	1,143	271	6.81	SPAdes
		172	358	130	6.99	ABySS^*^
	Illumina + PacBio	122	3,522	3,522	6.91	SPAdes
	PacBio-only	12	2,261	1,340	6.94	SMRTanalysis v 2.0^b^
	PacBio-only	3	6,349	6,349	6.88	SMRTanalysis v 2.2
	PacBio-only	**1**	**6,878**	**6,878**	**6.87**	**Manual Finishing**
*Clostridium pasteurianum* ATCC 6013	Illumina	6	4,108	4,108	4.36	SPAdes^*^
		101	207	73	4.35	ABySS
	Illumina + PacBio	9	4,022	4,022	4.36	SPAdes
	PacBio-only	**2**	**4,374**	**4,374**	**4.39**	**SMRTanalysis v 2.2**

Best assemblies shown in bold. The best draft assembly achieved with only the Illumina data are marked with ^*^.

a Additional numbers shown in brackets correspond to the extra-chromosomal plasmid DNA.

b *Assemblies performed prior to the availability of SMRTanalysis version 2.2. Prior assemblies are included to describe the effectiveness of algorithm improvement on genome assembly using the same data*.

Long read sequencing platforms are criticized for their frequent (~15%), but random errors in the PacBio platform can be corrected by using high (>100x) sequence coverage and/or Illumina data. However, uniform sequence coverage across the entire genome is not guaranteed and low coverage regions are prone to base-call errors. Assembly polishing is a crucial step to obtain accurate consensus sequence and facilitate downstream applications. Two assembly base-call correction algorithms applied in this study are Quiver (correction using PacBio reads) and Pilon (correction using Illumina reads) while iCORN (Otto et al., 2010) is another alternative. The default HGAP protocol is implemented with a single round of Quiver polishing and we applied additional rounds of Pilon correction for further assembly quality improvements. The majority of the base-call errors corrected by Pilon were insertions-deletions (indels) (Table 4 and Tables S11–S14), which were responsible for the frameshift mutations and correspond to altered Open Reading Frame (ORF) predictions. To validate the accuracy of Pilon calls, 47 random Pilon corrections across four finished genomes were verified by PCR and Sanger sequencing. Our results indicate that 40 of the 47 (~85%) tested corrections by Pilon were accurate and supported by two (forward and reverse) high quality Sanger reads. The remaining seven suggested Pilon modifications were ruled out based on lack of support from analysis of Sanger data.

**Table 4:** Summary of Pilon call verification by Sanger sequencing.

**Genome**	Total number of SNP^*^ verified by Sanger	**Total number of correct calls**	**Total number of incorrect calls**
*Pelosinus fermentans* JBW45	11	11	0
*Clostridium thermocellum* AD2	22	17	5
*Pelosinus* sp. UFO1	6	4	2
*Halomonas* sp. KO116	8	8	0
Total	47	40	7

* *SNP refers to polymorphisms as well as indels. 19 of 47 SNP calls were indels while 1 of 7 incorrect SNP calls was indel*.

Further evaluation of Pilon corrections was performed by measuring the changes in the protein coding potential and positive/negative influence on gene calling accuracy (Section S1). In most cases, Pilon corrections improved the protein coding potential by predicting longer ORFs, joined ORFs (previously split genes were joined together to represent single long ORF), and few novel ORFs (Tables S11–S14). Certain Pilon corrections resulted in split ORFs (previously longer ORF were split into two ORFs), but such cases were comparatively fewer than the number of improved ORFs. Moreover, most of the changed ORFs were associated with improved BLASTN results (e-value, percent similarity, percent identity, and subject length) suggesting enhanced gene-calling accuracy. To summarize, there were total 314 modifications suggested by Pilon across four finished genomes, of which 154 (49%) have resulted in improved protein coding potential (longer/joined/novel ORFs), 38 (12%) were associated with split/shorter ORFs while 122 (38%) had no change. Considering the BLASTN results, 183 (58%) corrections have a positive influence on gene calling accuracy, 35 (11%) corrections deteriorated the BLASTN results while 96 (31%) had no changes. Pilon is a useful tool for *in silico* genome refinement and recommended when Illumina data is available.

Another important aspect of finished genome sequences is an accurate representation of a circular chromosome. Automatically finished assemblies generated through HGAP often have (duplicated) overlapping ends which need to be trimmed off for the final assembly. This could be achieved using the circulator (Hunt et al., 2015) software which performs automated assembly circularization and sets the *dnaA* gene as the starting position. In this study, assembly circularization was performed manually through a read mapping and alignment approach before the availability of circlator software. Later, a comparison of circlator and manual assemblies was performed and results were similar (data not shown). Therefore, for future projects, the application of circlator software followed by a careful inspection of the trimmed regions is recommended.

## Conclusions

In this study, we present an effective manual finishing approach targeted toward near-finished microbial genome assemblies. The importance of genome polishing steps is demonstrated through its positive influence on gene calling accuracy and improved protein coding potential, which will be useful to others looking to improve long-read assemblies. Assessment of Illumina gaps confirmed previous findings that repetitive rRNA operons are major contributors to fragmented short-read assemblies. For PacBio assemblies, our initial hypothesis that structural features of DNA might affect the PacBio sequence coverage leading to assembly gap was not accepted. However, we demonstrated that certain biological features such as presence of active transposons, plasmid sequences, and phage integration are possible reasons for assembly fragmentation. Additionally, DNA regions flanking the PacBio gap sequences showed high degrees of similarity with other loci and are likely contributors to incomplete PacBio assemblies in this dataset. The PacBio gap sequences in this study are attributed to a cumulative effect of various aspects of repetitive DNA content and biological features for specific genomes. Despite a few limitations, long reads from third-generation sequencing, in this case from the PacBio platform, are particularly advantageous for generating *de novo* microbial genome assemblies. Our datasets and analyses will aid future efforts to better understand and overcome unassembled DNA from PacBio assemblies.

## Author contributions

SU designed the study, performed, and contributed to all the experiments and analyses and wrote the manuscript draft; DK extracted genomic DNA, performed Illumina sequencing, and assisted with PCR and Sanger sequencing; RH contributed to study design and edited the manuscript; SB contributed to study design, assisted with draft writing, and editing. All authors reviewed and approved the manuscript.

### Conflict of interest statement

The authors declare that the research was conducted in the absence of any commercial or financial relationships that could be construed as a potential conflict of interest.
