# Supplementary material for: A Case Study into Microbial Genome Assembly Gap Sequences and Finishing Strategies
Source: Front Microbiol. 2017 Jul 18;8:1272. doi: 10.3389/fmicb.2017.01272 (PMC5513972; doi:10.3389/fmicb.2017.01272)
Supplement: Supplementary file 1 [file DataSheet1.pdf]

# A case study into microbial genome assembly gap sequences and finishing strategies

Sagar M. Utturkar[1,] Dawn M. Klingeman[2,3], Richard A. Hurt Jr.[2], and Steven D. Brown[1,2,3,*]

[1]Graduate School of Genome Science and Technology, University of Tennessee, Knoxville, TN 37919, USA

[2]Biosciences Division, Oak Ridge National Laboratory, Oak Ridge, TN 37831, USA

[3]BioEnergy Science Center, Oak Ridge, Tennessee, USA

[*]To whom correspondence should be addressed. Tel: +865-576-2368; Email: brownsd@ornl.gov

Present Address: S. Utturkar, Bioinformatics Core, Purdue University, West Lafayette, Indiana 47907, USA

**Supplementary section S1**

**Manual finishing overview:**
Requirements for the manual finishing approach described here are Illumina and PacBio data, and a near-finished genome assembly (containing < 10 contigs). Manual finishing starts with bioinformatics steps in which contigs from the multiple draft/hybrid assemblies are mapped to the near-finished genome assembly using "Map to Reference" module in Geneious followed by the manual inspection to derive putative contig extensions. Contig extension were performed when contig overlap was minimum 10 kb and with less than 1% mismatches. Re-assembly of contigs with extended ends (super-assembly) is performed to derive longer consensus sequence (super-contig) using "*de novo* assembly" module in Geneious. Bioinformatically derived contig extensions and assembled consensus are validated by PCR and Sanger sequencing approach. During PCR, oligonucleotide primers designed which are flanking the contigs overlap/extension and validation occurs when PCR amplified products of the predicted size. In the case of large predicted PCR products (> 3 kb), an additional set of internal oligonucleotide primers were designed to amplify the end regions.

**PCR and Sanger sequencing protocol:**
PCR reactions were performed using a Phusion High-Fidelity PCR Kit (New England Biolabs, Ipswich, MA) following the manufacturer's protocol. PCR product purification was performed using MinElute PCR purification kit (Qiagen) following the manufacturer's protocol. Sanger sequencing of purified PCR products was performed at the University of Tennessee Genomics Core facility using ABI 3730 Genetic Analyzer Instrument (Life Technologies). Sanger reads were quality trimmed and aligned to the bioinformatically derived consensus sequence using Geneious software to verify the accuracy and assembly contiguity.

The PCR/Sanger sequencing based validations were particularly challenging when there was no bioinformatics evidence available for the contig extension/overlap. In such case (e.g. finishing for *B. cellulosolvens* DSM 2933), several experimental modifications were applied to standard PCR which includes:

a. Use of strand-displacing *Pfu* DNA polymerase for uncoiling of double-stranded DNA
b. Use of nucleotide analogue 7-deaza-2′-dGTP instead of standard guanine nucleotide which forms comparatively weaker bond with cytosine nucleotide and helps to break strong DNA hairpin structures[1].
c. Determination of appropriate PCR conditions through multiple experimental parameter optimizations (primer concentrations, annealing temperatures, and extended time for DNA denaturation) as described previously[1].

**Manual finishing of *C. thermocellum* AD2**
The best automatic assembly for strain AD2 using PacBio data contained 10 contigs. Mapping of the draft assemblies (generated by SPAdes and ABySS) to the 10 contig reference sequence derived extension to the 7 contigs. Super-assembly of all 10 contigs (including 7 extended contigs) generated 4 super-contigs. The longest super-contig (AD2_SC1) was of size 2.06 Mb and derived from the assembly of three extended draft contigs (**Figure S1**). Validation of two putative overlaps (AD2_overlap1 and AD2_Overlap2) between three extended draft contigs and accuracy of the resulting consensus sequence (AD2_SC1) was performed by PCR and Sanger sequencing approach (**Figure S1**).

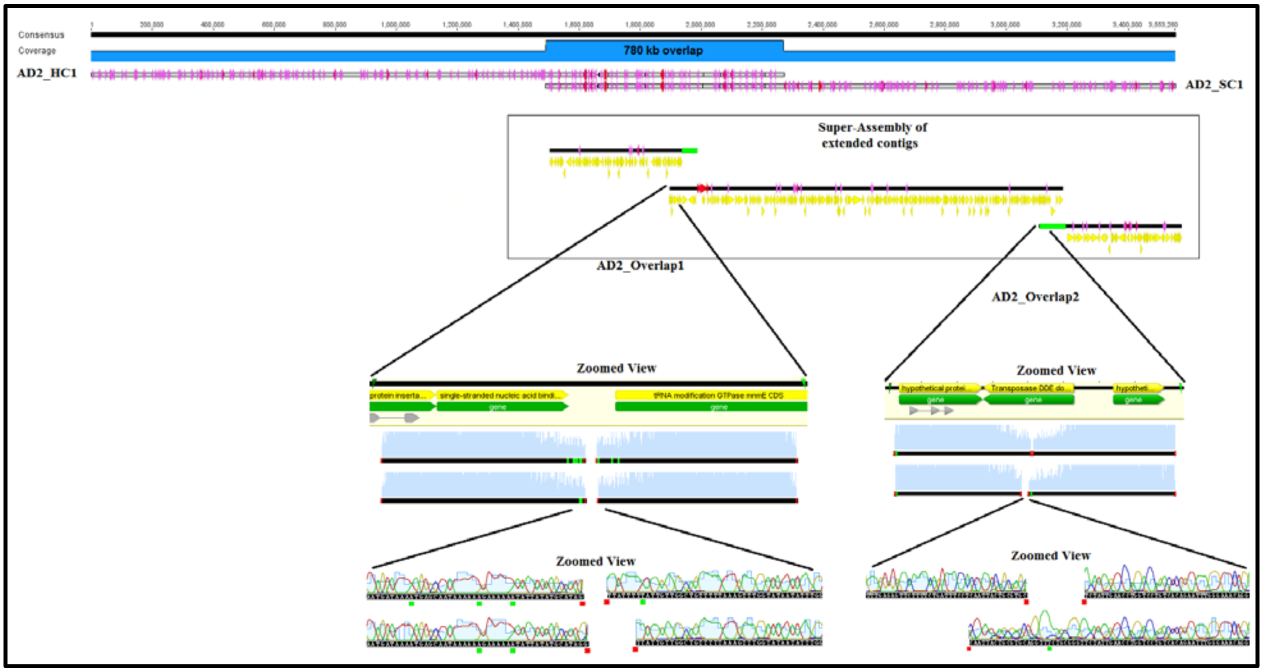


**Figure S1** - **AD2 genome finishing overview.** Top row shows the 3.5 Mb consensus derived from the super assembly of two longest contigs AD2_SC1 and AD2_HC1 with 780 kb overlap. The AD2_SC1 contig was derived from super assembly of three contigs with extended ends (highlighted in green). The super assembly of AD2_SC1 was validated by PCR and Sanger sequencing of contigs overlaps AD2_overlap1 and AD2_overlap2. Zoomed view shows the corresponding annotations, and alignment of Sanger reads.

During manual inspection of mapping results (Illumina draft contigs mapped to PacBio assembly), a long 2.27 Mb contig (AD2_HC1) was identified in the SPAdes hybrid assembly. The two longest contigs (AD2_SC1 and AD2_HC1) represented two opposite ends of the genome and super-assembly derived a 3.5 Mb consensus sequence with ~780 Kb overlapping sequence (**Figure S1**). The 780 Kb overlapping region contained only a handful of base-pair mismatches which were manually corrected by mapping of the Illumina reads. The expected genome size for the AD2 genome was in the ~3.5 Mb range. However, dot plot and other tests for genome circularity could not confirm the circular nature of the consensus sequence. Therefore, additional PCR amplification reactions were performed to extend the ends of the 3.5 Mb consensus sequence. Sanger sequencing of a ~1 kb purified PCR product revealed the sequence for this gap region (AD2_Gap1) comprising total 1,069 bp. After gap-closure, a 3,554,860 bp circular genome sequence for strain AD2 was obtained. Annotation of the 1,069 bp gap sequence corresponded to gene models for repetitive transposon DNA - “transposon mutator type CDS”. Sequences derived for (AD2_overlap1, AD2_Overlap2 and AD2_Gap1) constituted the gaps present within the PacBio assemblies.

**Manual finishing of *B. cellulosolvens* DSM 2933**

The best assembly for *B. cellulosolvens* DSM 2933 using PacBio data contained 3 contigs. Scaffolding through AHA protocol[2] could not improve assembly any further. Mapping of the draft or hybrid contigs to a three contig reference could not extend any contig ends. However, super-assembly of 3 contigs detected a 6.7 kb overlap (BC_overlap1) between contigs BC_C1 and BC_C3. Validation of contig overlap and consensus sequence was performed through PCR and Sanger sequencing (**Figure S2**).

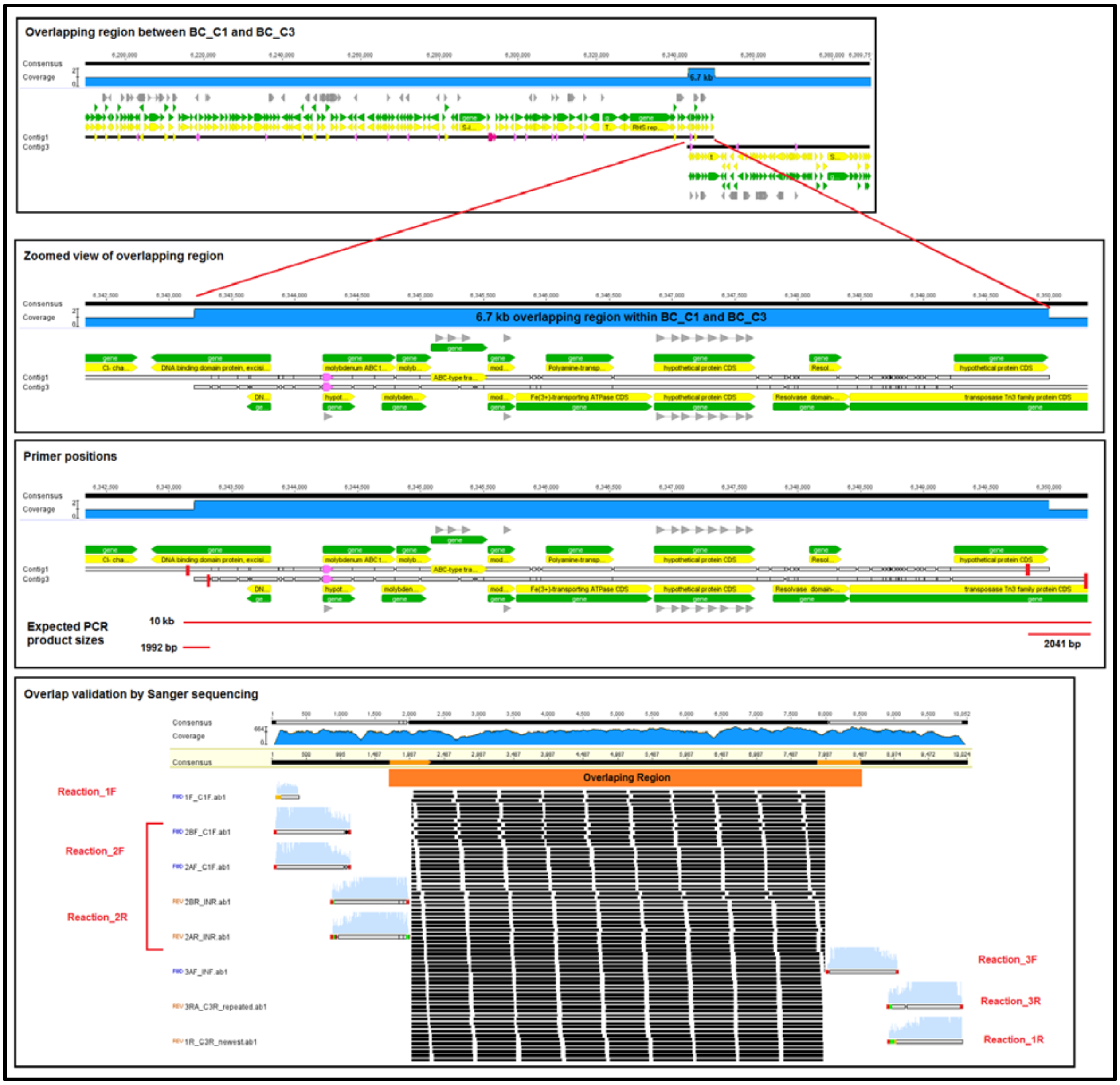


**Figure S2** - ***B. cellulosolvens* genome finishing overview.** Super assembly of contigs BC_C1 and BC_C2 with 6.7 kb overlap. Zoomed view shows associated annotations, primer positions, expected PCR product sizes, and overlap validation by high-quality Sanger and Illumina reads.

The remaining contig (BC_C2) could not be joined together using bioinformatics approaches and indicated presence of an unknown gap (BC_Gap1). Oligonucleotide primers were designed on either ends of the two contigs followed by the multiple rounds of PCR with various primer combinations. A successful PCR amplification and separation of PCR products on agarose gel obtained a clean band at ~400 bp location. Sanger sequencing uncovered the 406 bp sequence for the BC_Gap1 (**Figure S3**).

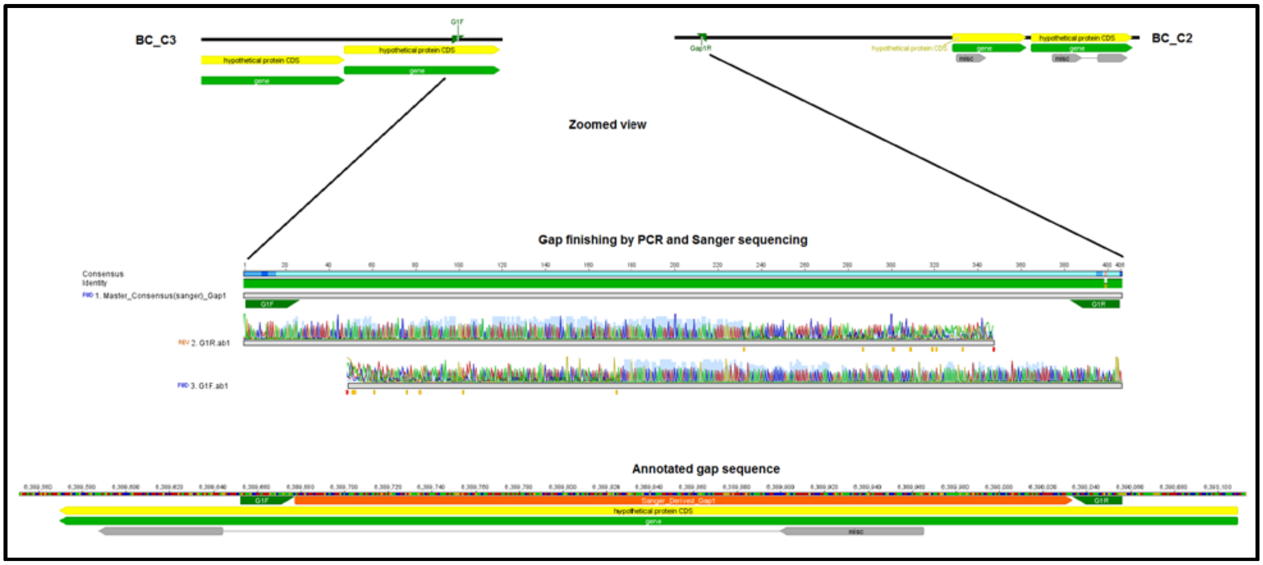


**Figure S3** - ***B. cellulosolvens*** **gap finishing overview.** Sequence for the gap existed within contigs BC_C3 and BC_C2 was derived by PCR and Sanger sequencing approaches. Zoomed view shows primer positions, alignment of high-quality Sanger reads, and post-finishing annotation of gap sequence.

In this study, the BC_Gap1 region was the most difficult region to resolve by PCR/Sanger approach and perhaps for the multiple reasons. First of all, there was no bioinformatics based evidence available in terms of overlapping contigs, the order and orientation of adjoining contigs was unknown, and other genomes from the same lineage had low (below 90%) average nucleotide identity making predictions about contig order difficult. The closure of BC_Gap1 permitted a single contig assembly for the strain DSM 2933, however; further finishing approaches to obtain the circular chromosome were unsuccessful and genome was deposited at Genbank with near-finished status. Two sequences derived through manual finishing (BC_overlap1 and BC_Gap1) constituted the gaps present within the PacBio assemblies.

**Note:** In current manual finishing protocol, we have extensively used commercial licensed software Geneious (Biomatters, Auckland, New Zealand)[3] which was convenient to use. However, all the functionalities and analysis can be also performed through various open source tools may be without graphical user interface. Mapping reads to reference can be achieved using bowtie2[4], mapping of contigs to reference can be performed through mauve plugin[5], assembly of contigs can be performed through CAR[6] or other assemblers and pairwise alignment of sequences through MUSCLE aligner[7]. Read quality assessment and trimming can be performed through FASTQC (http://www.bioinformatics.babraham.ac.uk/projects/fastqc/) and FASTX (http://hannonlab.cshl.edu/fastx_toolkit/) tools. Therefore, the availability of the commercial tool does not limit application of current protocol.

**Assembly validation protocols:**

1. <u>Differences between protein coding potential of any two assemblies</u>
   Reciprocal BLASTP [8] analysis was performed using proteins predicted from the draft and the finished genome assemblies to gain insights into potential protein encoding differences. The blast hits across two genomes were analyzed using custom script to identify protein sequences which are same across two genomes, longer proteins, and shorter protein. Generally, the finished genome assembly was used as the reference to identify same, shorter, or longer proteins.

2. <u>Estimation of positive/negative influence of Pilon change</u>
   The BLASTN analysis of predicted genes before and after Pilon change was performed against the NCBI non-redundant database. The effect of Pilon call on BLASTN results was determined in terms of e-value, or percent similarity, or percent identity or subject hit length. The Pilon corrections which improved e-value cutoffs, percent similarity or percent identity scores and increased subject hit lengths are considered as having positive influence on gene-calling accuracy. On the other hand, Pilon corrections which diminished the significance of BLASTN results (e-value cutoffs, percent similarity or percent identity scores or decreased subject hit lengths) are considered to have negative influence on gene-calling accuracy. Positive and negative influence of Pilon correction is indicated by the “Yes” and “No” values, respectively in **supplementary table S11-S14**.

**Assembly protocols:**

1. Assembly of Illumina data using SPAdes.

```
spades.py \
-k 21,33,55,77,99,127 \
--pe1-12 < trimmed_reads_12.fastq>
--careful -o spades_out
```

2. Assembly of Illumina data using ABySS.

```
abyss-pe k=31 in='<trimmed_reads_12.fastq>' name=31 > std.out 2> std.err
abyss-pe k=49 in='<trimmed_reads_12.fastq>' name=49 > std.out 2> std.err
abyss-pe k=57 in='<trimmed_reads_12.fastq>' name=57 > std.out 2> std.err
abyss-pe k=61 in='<trimmed_reads_12.fastq>' name=61 > std.out 2> std.err
abyss-pe k=63 in='<trimmed_reads_12.fastq>' name=63 > std.out 2> std.err
```

3. Hybrid Assembly of Illumina and PacBio data using SPAdes.

```
spades.py \
-k 21,33,55,77,99,127 \
--pe1-12 < trimmed_reads_12.fastq>
--pacbio <filtered_Pacbio_reads.fasta>
--careful -o spades_out
```

4. Assembly polishing using Pilon.

```
Pilon --genome genome.fasta \
--frags frags.bam \
--changes
```

**References:**


1. Hurt, R.A., Brown, S.D., Podar, M., Palumbo, A.V. & Elias, D.A. Sequencing intractable DNA to close microbial genomes. *PLoS One* **7**, 7 (2012).
2. Bashir, A. et al. A hybrid approach for the automated finishing of bacterial genomes. *Nat. Biotechnol.* **30**, 701-707 (2012).
3. Kearse, M. et al. Geneious Basic: an integrated and extendable desktop software platform for the organization and analysis of sequence data. *Bioinformatics* **28**, 1647-1649 (2012).
4. Langmead, B. & Salzberg, S.L. Fast gapped-read alignment with Bowtie 2. *Nat. Methods* **9**, 357-359 (2012).
5. Darling, A.C., Mau, B., Blattner, F.R. & Perna, N.T. Mauve: multiple alignment of conserved genomic sequence with rearrangements. *Genome Res.* **14**, 1394-1403 (2004).
6. Lu, C.L., Chen, K.T., Huang, S.Y. & Chiu, H.T. CAR: contig assembly of prokaryotic draft genomes using rearrangements. *BMC Bioinformatics* **15**, 381 (2014).
7. Edgar, R.C. MUSCLE: multiple sequence alignment with high accuracy and high throughput. *Nucleic Acids Res.* **32**, 1792-1797 (2004).
8. Altschul, S.F. et al. Gapped BLAST and PSI-BLAST: a new generation of protein database search programs. *Nucleic Acids Res.* **25**, 3389-3402 (1997).
